# Spatially confined multi-enzyme cascade nanoreactor for portable and real-time pesticide detection

**DOI:** 10.1186/s12951-026-04677-8

**Published:** 2026-06-11

**Authors:** Wei Zhang, Jia Shu, Chonghai Gong, Song Zhou, Hanting Wang, Kunyang Feng, Dongmei Liu, Rui Chen, Juan Li, Zhengwei Zhang, Siqiao Li

**Affiliations:** 1 School of Basic Medical Sciences, Chongqing Medical University, Chongqing, 400016 China; 2 Ophthalmology Medical Center, Chongqing Key Laboratory for the Prevention and Treatment of Major Blinding Eye Diseases, Chongqing Branch (Municipality Division) of National Clinical Research Centre for Ocular Diseases, The First Affiliated Hospital of Chongqing Medical University, Chongqing, 400016 China; 3 College of Pharmacy, Chongqing Medical University, Chongqing, 400016 China; 4 Key Laboratory of Clinical Laboratory Diagnostics (Ministry of Education), College of Laboratory Medicine, Chongqing Medical University, Chongqing, 400016 China

**Keywords:** Multi-enzyme cascade nanoreactor, Spatial confinement, Dual-mode visual biosensing, Organophosphorus pesticides

## Abstract

Multi-enzyme cascade reactions offer powerful amplification and accelerated biotransformation, yet their implementation in free-solution systems is limited by enzyme instability, diffusion losses, and low coupling efficiency. Here, we reported a spatially confined multi-enzyme cascade nanoreactor (AChE/CHO/HRP@MSN) constructed by co-immobilizing acetylcholinesterase (AChE), choline oxidase (CHO), and horseradish peroxidase (HRP) within amino-functionalized mesoporous silica nanoparticles (MSN-NH_2_). The ordered mesoporous architecture provides high loading capacity, efficient substrate channeling, and enhanced structural stability, enabling tightly coupled multi-step biocatalysis even in complex environments. The AChE/CHO/HRP@MSN platform supports dual-mode colorimetric and fluorescent sensing of organophosphorus pesticides (OPs) and enables real-time monitoring of pesticide degradation in plants. By coupling with a smartphone-readable hydrogel matrix, the system enables portable signal output and on-site visual monitoring of OP residues. This spatially organized, cascade-driven strategy not only addresses longstanding limitations in multi-enzyme system design but also provides a versatile framework for programmable biosensing in complex environments.

**Graphical Abstract:**

**Figure:**
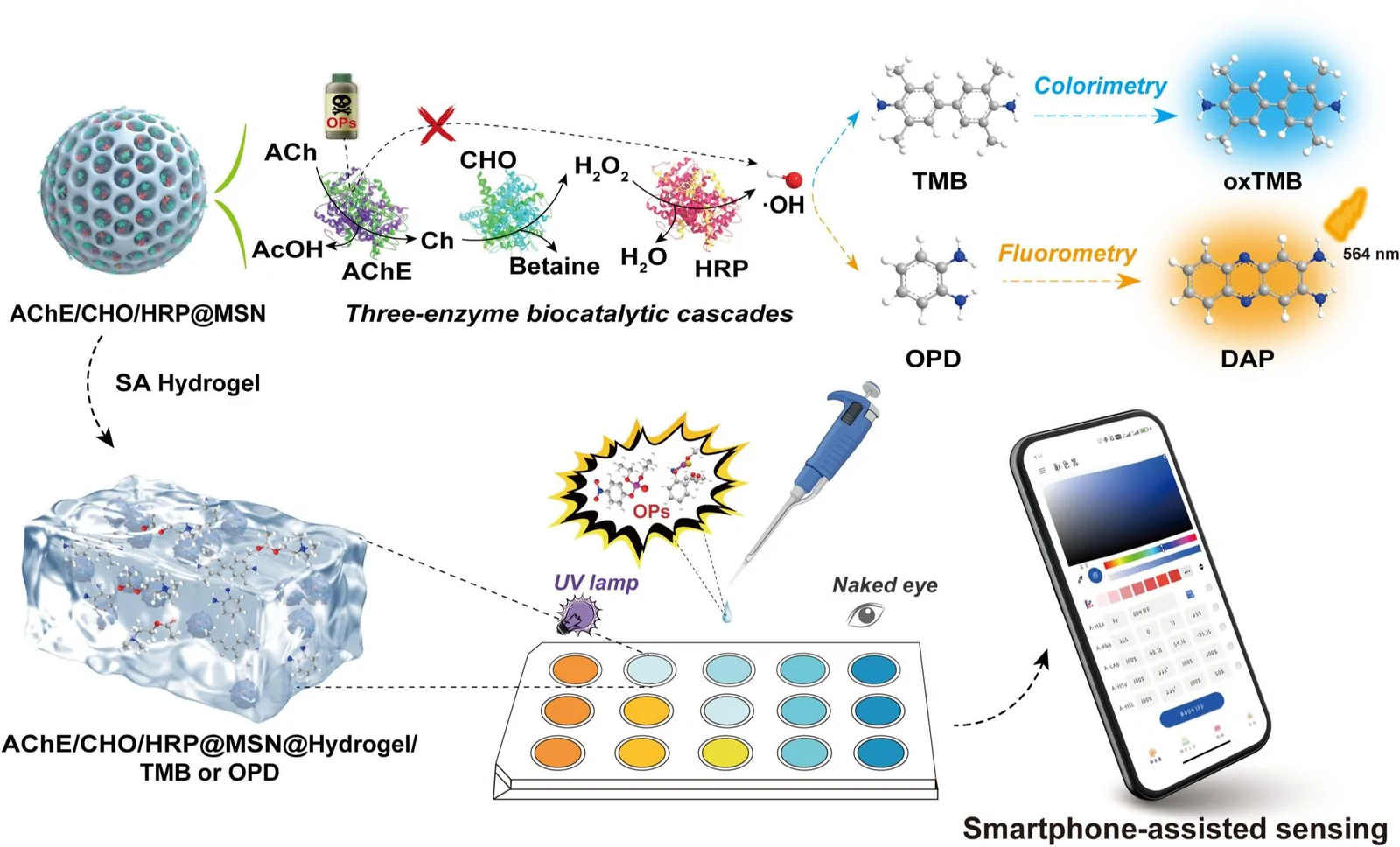

**Supplementary Information:**

The online version contains supplementary material available at 10.1186/s12951-026-04677-8.

## Introduction

Enzyme cascades enable efficient intermediate transfer and signal amplification, but free multi-enzyme systems often suffer from poor stability, kinetic mismatch, and diffusion loss, limiting their practical performance in biosensing and bioconversion [1–3]. Enzyme immobilization represents a powerful and widely adopted strategy to enhance the catalytic performance, stability, and reusability of enzyme systems. By anchoring enzymes onto solid supports through physical adsorption, covalent bonding, or spatial confinement, immobilization creates a defined microenvironment that protects the biocatalyst from external stressors such as temperature fluctuations, pH instability, and organic solvents [4, 5]. More importantly, immobilization facilitates the formation of localized spatiotemporal architectures, where substrate channeling between sequential enzymes is promoted and mass-transfer resistance is reduced. These spatially confined reaction environments markedly enhance the kinetics and coupling efficiency of multi-enzyme cascade reactions, which are otherwise limited by intermediate diffusion and enzymatic incompatibility in free systems [6, 7].

A variety of materials, including metal–organic frameworks, porous polymers, and hydrogels, have been explored as enzyme immobilization matrices because of their tunable porosity, functional interfaces, and structural versatility [8]. Porous carriers are particularly attractive for cascade systems, as their confined architectures can enrich substrates, retain intermediates, and spatially organize multiple enzymes within a restricted domain [9]. Hydrogels further provide a hydrated and biocompatible environment that helps preserve enzymatic activity and supports portable and in situ analytical applications. However, current immobilization platforms still suffer from several limitations, including low enzyme loading, weak enzyme–support interactions, poor long-term structural stability, and insufficient microenvironmental regulation. These shortcomings often lead to enzyme leakage, activity loss, and reduced sensing reliability in complex matrices [10]. Therefore, developing advanced immobilization platforms that integrate high enzyme affinity, structural robustness, efficient substrate/intermediate transport, and microenvironmental responsiveness remains essential for the practical implementation of multi-enzyme cascade systems.

Mesoporous materials, characterized by their high specific surface area, tunable pore size, and excellent pore connectivity, exhibit superior adsorption capacity and mass transfer efficiency, making them highly attractive carriers for enzyme immobilization and catalytic reactions [11–13]. By precisely engineering the pore architecture through physical templating or chemical modification, enzymes can be efficiently immobilized or anchored within the mesoporous matrix, thereby promoting localized catalytic reactions and enabling rapid signal transduction. These materials not only facilitate high enzyme loading and structural stabilization, but also significantly improve enzymatic activity retention and kinetic performance under operational conditions [14, 15]. Among them, mesoporous silica nanoparticles (MSNs) represent a benchmark due to their adjustable pore structures (2–50 nm), excellent chemical/thermal stability, biocompatibility, and versatile surface functionalization [16, 17]. The silanol-rich surface of MSNs enables covalent or non-covalent enzyme anchoring, while the rigid yet porous structure minimizes enzyme leaching and enhances resistance to environmental fluctuations. These advantages collectively position MSNs as an ideal scaffold for constructing confined multi-enzyme cascade systems, wherein spatial proximity and substrate channeling can be finely regulated. Despite these advantages, MSN-based systems have rarely been engineered into fully integrated, multi-modal catalytic platforms capable of real-time and visual detection of toxic analytes in complex environments.

**Scheme 1:**
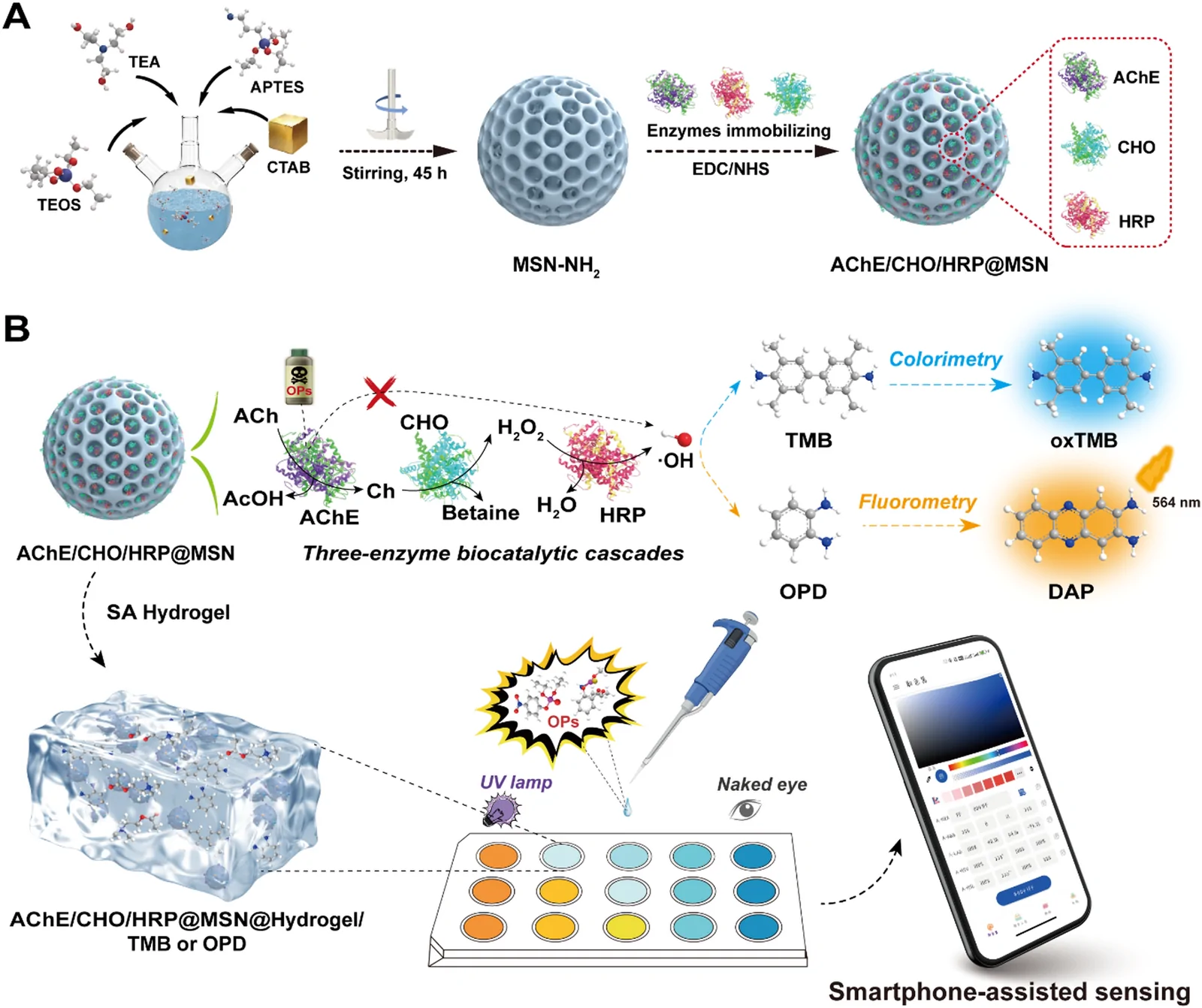
(**A**) Schematic of the fabrication of the spatially confined AChE/CHO/HRP@MSN nanoreactor. (**B**) The construction of smartphone-assisted AChE/CHO/HRP@MSN@Hydrogel platform for on-site OP detection

Inspired by the aforementioned advances, we developed a spatially confined multi-enzyme cascade nanoreactor (AChE/CHO/HRP@MSN) by co-immobilizing three synergistic enzymes—acetylcholinesterase (AChE), choline oxidase (CHO), and horseradish peroxidase (HRP)—within a mesoporous silica framework (Scheme 1). The ordered MSN architecture provides high loading capacity and short diffusion pathways, enabling efficient substrate channeling along the cascade. In this system, AChE hydrolyzes acetylcholine (ACh) to choline, CHO oxidizes choline to generate hydrogen peroxide (H_2_O_2_), and HRP converts H_2_O_2_ into highly reactive hydroxyl radicals (·OH) that oxidize 3,3’,5,5’-tetramethylbenzidine (TMB) and o-phenylenediamine (OPD), producing distinct colorimetric and fluorescent signals. Organophosphorus pesticides (OPs) selectively inhibit AChE, attenuating the cascade and enabling quantifiable suppression of both optical outputs. The resulting nanoreactor exhibits high enzyme-activity retention, excellent operational stability, and strong anti-interference capacity under complex conditions. Using paraoxon as a model analyte, we further integrated AChE/CHO/HRP@MSN into a hydrogel matrix to create a smartphone-assisted, portable biosensor for on-site visual OP detection. Notably, the platform supports real-time monitoring of pesticide degradation in actual plant samples, offering a reliable analytical tool for dynamic residue tracking and environmental risk assessment. This work presents a robust strategy for the rational construction of localized multi-enzyme cascade systems, and offers a promising foundation for developing high-performance biosensors aimed at environmental health monitoring and food safety evaluation.

## Materials and methods

### Reagents

AChE, CHO, HRP, and HAc-NaAc buffer were supplied by Yuanye Biotechnology Co., Ltd. (Shanghai, China). ACh and bovine serum albumin (BSA) were purchased from Sigma-Aldrich Reagent Co., Ltd. (USA). Acetylthiocholine chloride (ATCh), OPD, and sodium alginate (SA) were purchased from Macklin Biochemical Co., Ltd. (Shanghai, China). Choline chloride (Ch), 5,5-dithiobis(2-nitrobenzoic acid) (DTNB), TMB, 1-ethyl-3-(3-(dimethylamino)propyl)carbodiimide hydrochloride (EDC), N-hydroxysuccinimide (NHS), cetyltrimethylammonium bromide (CTAB), triethanolamine (TEA), tetraethyl orthosilicate (TEOS), and (3-aminopropyl)triethoxysilane (APTES) were obtained from Aladdin Chemistry Co., Ltd. (Shanghai, China). Fluorescein isothiocyanate isomer (FITC) was purchased from Xi’an Qiyue Technology Co., Ltd. (Shanxi, China). Dichlorvos, dimethoate, phosalone, paraoxon, isocarbophos, phenthoate, parathion, methyl parathion, methamidophos, acephate, malathion, and glyphosate were purchased from Ehrenstorfer (Germany). Ethanol, acetone, isopropanol, cyclohexane, dichloromethane, and chloroform were purchased from Chongqing Chuandong Chemical Co., Ltd. (Chongqing, China). All solvents and chemicals were procured from commercial sources and used without further purification.

### Apparatus

Ultraviolet–visible (UV–vis) absorption spectra were recorded using a HITACHI UH-5300 spectrophotometer (Hitachi, Japan). Fluorescence spectra were acquired on a HITACHI F-4700 fluorescence spectrophotometer (Hitachi, Japan). Scanning electron microscopy (SEM) images were acquired on an S-4800 electron microscope (Hitachi, Japan). Transmission electron microscopy (TEM) images were acquired using an FEI Tecnai G2 12 instrument (Thermo, USA). Energy dispersive spectroscopy (EDS) was performed using an FEI Tecnai G2 F20 instrument (Thermo, USA). Powder X-ray diffraction (XRD) was performed with a D8 ADCANCE X-ray diffractometer (Bruker, Germany) with Cu Kα radiation (λ = 1.5406 Å). X-ray photoelectron spectroscopy (XPS) was performed on a Thermo Escalab 250XI X-ray photoelectron spectrometer (Thermo, USA) with an Al Kα excitation source. Zeta potential was measured with a Zetasizer nano ZS90 instrument (Malvern, UK). Fourier transform infrared (FT-IR) spectroscopy was performed using a Nicolet IS10 instrument (Nicolet, USA). N_2_ adsorption–desorption measurements were performed using an ASAP 2460 automatic physical adsorption instrument (Micro, USA).

### Synthesis of amino-functionalized MSNs (MSN-NH_2_)

MSN-NH_2_ were synthesized as per a previous method with slight modifications [18]. CTAB (1 g) and TEA (50 µL) were dissolved in 20 mL of deionized water and stirred at 60 ℃ for 1 h. Subsequently, a mixture of 1.5 mL TEOS and 6 mL cyclohexane was added, and stirring was continued for 24 h. At the end of the reaction, the precipitate was centrifuged and dissolved in 30 mL acetone and stirred at 70 ℃ for 12 h to remove the template. After centrifugation, the precipitate was dissolved in 30 mL ethanol, and 2 mL APTES was added and stirred at 60 ℃ for 8 h. Finally, MSN-NH_2_ were collected via centrifugation and dried at 50 ℃. The final product was stored at 4 ℃ for further use.

### Synthesis of the AChE/CHO/HRP@MSN nanoreactor

The AChE/CHO/HRP@MSN nanoreactor was synthesized via a modified solvothermal process as per the literature [19]. MSN-NH_2_ (0.5 mg) was thoroughly dispersed in 0.3 mL of a phosphate-buffered saline (PBS) solution. Then, 50 µg AChE, 50 µg CHO, 50 µg HRP, 50 µL of EDC (2 mg/mL) solution, and 50 µL of NHS (1 mg/mL) solution were successively added into the aforementioned PBS solution. After the reaction was maintained at room temperature for 2 h, 50 µL of BSA (10%) solution was added and stirred for 2 h. Finally, the sample was collected via centrifugation and the resulting product was stored at 4 °C for further use.

### Measurement of enzymatic activities in the AChE/CHO/HRP@MSN nanoreactor

The catalytic activities of AChE, CHO, and HRP in the AChE/CHO/HRP@MSN nanoreactor were measured individually. The concentrations of free enzymes were consistent with the corresponding enzymes in AChE/CHO/HRP@MSN on the basis of the calculated loading capacity, and then, the catalytic activities of the free and immobilized enzymes were measured. The catalytic activities of AChE, CHO, and HRP were evaluated by applying Eqs. (1) and (2), (3) and (4), and (4), respectively.

1 $$\begin{aligned} \mathrm{Acetylthiocholine} + \mathrm{H_2O} \xrightarrow{\mathrm{AChE}} \mathrm{Thiocholine} + \mathrm{Acetate\ acid} \end{aligned}$$

2 $$\begin{aligned} \mathrm{Thiocholine} + \mathrm{DTNB} \rightarrow \mathrm{TNBA} + \mathrm{Mixed\ disulfide} \end{aligned}$$

3 $$\begin{aligned} \mathrm{Choline} + \mathrm{O_2} \xrightarrow{\mathrm{CHO}} \mathrm{Betaine} + \mathrm{H_2O_2} \end{aligned}$$

4 $$\begin{aligned} \mathrm{H_2O_2} + \mathrm{TMB} \xrightarrow{\mathrm{HRP}} \mathrm{H_2O} + \mathrm{TMBox} \end{aligned}$$

The kinetic parameters (K_m_ and V_max_) were calculated by applying Michaelis–Menten equation:

*1/V = (Km/Vmax) (1/[S] + 1/Vmax)*

where [S] stands for the concentration of substrate, and V stands for the reaction rate at this concentration of the substrate.

### Dual-mode biosensing for OPs

The AChE/CHO/HRP@MSN solution (10 µL, 1 mg/mL) and 10 µL of different concentrations of OPs solution (1–4000 ng/mL) were added to 96-well plates, and the same volume of ultrapure water was used as a blank control. After incubation at 37 °C for 10 min, 10 µL of the ACh solution (5 mM) was added and incubated for 40 min. Subsequently, 50 µL of the TMB solution (1 mM) and 100 µL of the HAc-NaAc buffer (pH = 6.5, 0.1 M) were added and incubated at 37 °C for 20 min, and the absorbance at 652 nm was measured using a UV–vis spectrophotometer. Under the same conditions, the TMB solution was replaced by the OPD solution (20 mM), and the fluorescence intensity at 564 nm was recorded using a fluorescence spectrophotometer after incubation at 37 ℃ for 40 min.

### Smartphone-assisted hydrogel device for on-site OP detection

SA (8 mg) was dissolved in 1 mL of ultrapure water and magnetically stirred at 60 °C for 15 min. A CaCl_2_ solution (20 µL, 6 mg/mL) was added and stirred continuously for 15 min. The mixture was transferred to a 96-well plate and refrigerated for 12 h to form the hydrogel. For OP detection, AChE/CHO/HRP@MSN was first immobilized in the hydrogel, and then, 40 µL of different concentrations of the OP solution (1–400 ng/mL) were added to the hydrogel and incubated at 37 ℃. Subsequently, 10 µL of ACh (5 mM) and 100 µL of the TMB/OPD solution (1 mM/20 mM) were added to produce a colored signal. After a specific time, the visible color of the hydrogel was captured using a smartphone camera under constant conditions, and the RGB values were extracted by utilizing the Color Picker app. The standard equation was obtained by linearly fitting the RGB values to the OP concentration.

### Detection of OPs in actual samples

The practicality of the constructed biosensing platform was assessed by detecting OPs in tap water, cabbages, and apples. Cabbage and apple samples were pretreated in accordance with LS/T 6139 − 2020: the samples were crushed and extracted via ultrasonic extraction using a solvent mixture of ethyl-acetate–methanol (95:5) containing 0.2 g/mL Na_2_SO_4_, and the supernatant was collected and dried at 45 °C. The dried samples were redissolved in pure water and measured as per the procedure described previously. Tap water samples were tested directly after filtration. The detection performance of AChE/CHO/HRP@MSN was further evaluated by adding different concentrations of paraoxon (50, 100, and 200 ng/mL) to the actual samples. In addition, pepper plants were purchased from the Chongqing plant market. The paraoxon standard solution (20 µL; 100 µg/mL) was uniformly sprayed onto the leaves and fruits of the pepper plants. Then, the plants were placed in a natural environment to simulate the degradation process of paraoxon. The residue of paraoxon was continuously monitored for 8 days. The leaves/fruits of the pepper plants were first pretreated in accordance with LS/T 6139 − 2020 and then tested by employing the aforementioned method.

## Results and discussion

### Synthesis and characterization of MSN-NH_2_ and AChE/CHO/HRP@MSN

**Fig. 1:**
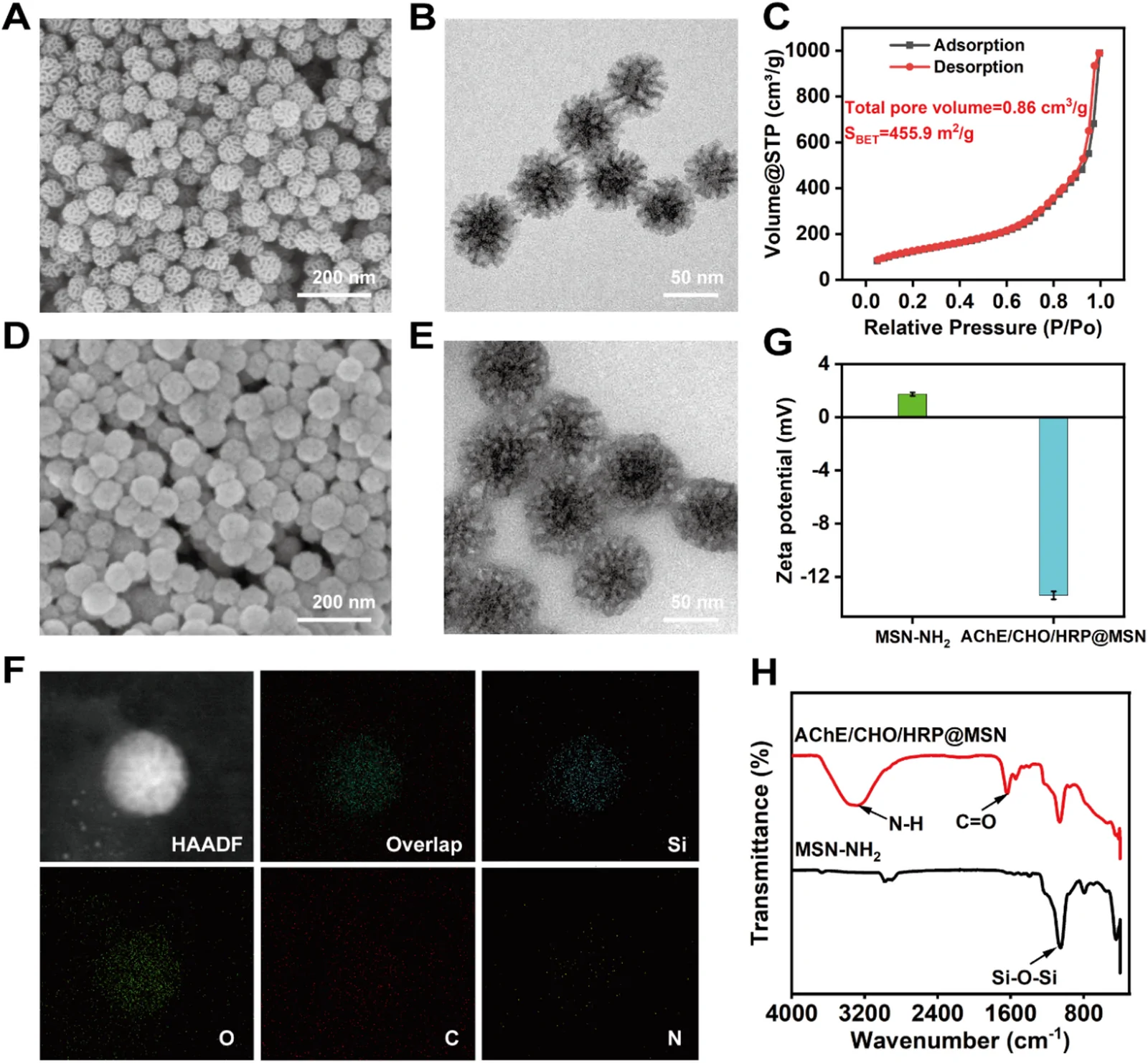
(**A**) SEM image of MSN-NH_2_. (**B**) TEM image of MSN-NH_2_. (**C**) N_2_ adsorption–desorption isotherms of MSN-NH_2_. (**D**) SEM image of AChE/CHO/HRP@MSN. (**E**) TEM image of AChE/CHO/HRP@MSN. (**F**) EDS mapping image of Si, O, C, and N distributions in AChE/CHO/HRP@MSN. (**G**) Zeta potential of MSN-NH_2_ and AChE/CHO/HRP@MSN. (**H**) FT-IR spectra of MSN-NH_2_ and AChE/CHO/HRP@MSN

The design and conduction of the multi-enzyme cascade system are illustrated in Scheme 1A. MSN-NH_2_ were first synthesized via a well-established templating method, using TEOS as the silica precursor and CTAB as the structure-directing agent. Surface amination was achieved through post-synthetic modification with APTES, resulting in well-defined mesoporous nanoparticles having uniform morphology and good dispersibility. The morphology, structure, and particle size distribution of MSN-NH_2_ were characterized by SEM and TEM (Fig. 1A and B). The synthesized particles exhibited a typical mesoporous architecture with an average diameter of approximately 60 nm and uniform dispersion. EDS confirmed the elemental composition and uniform spatial distribution of Si, O, and N within the MSN-NH_2_ framework (Fig. S1). The presence of Si and O revealed the successful formation of the silica matrix, whereas the homogeneous distribution of N indicated effective surface functionalization with amino groups. These results collectively confirmed the successful synthesis of MSNs-NH_2_ and their suitability as an enzyme immobilization carrier. The N_2_ adsorption–desorption isotherms displayed the typical mesoporous structural characteristics of MSN-NH_2_ (Fig. 1C). Brunauer–Emmett–Teller analysis revealed a high specific surface area of 455.9 m^2^/g and pore volume of 0.86 cm^3^/g. The average pore size, calculated by employing the Barrett–Joyner–Halenda method, was approximately 12.5 nm (Fig. S2). The high porosity and large surface area of MSN-NH_2_ provided abundant active sites and sufficient spatial capacity for efficient enzyme immobilization.

The elemental composition and chemical states of MSN-NH_2_ were comprehensively characterized by XPS. The survey spectrum confirmed the presence of C, N, Si, and O, consistent with the EDS results (Fig. S3A) [20]. High-resolution XPS spectra were further analyzed to determine the chemical bonding states of these elements. The C 1 s spectrum exhibited three distinct peaks at binding energies of 285.9, 285.4, and 284.7 eV, corresponding to C–O, C–N, and C–C/C=C bonds, respectively (Fig. S3B) [21]. The O 1 s spectrum showed two characteristic peaks at 532.5 and 532.8 eV, which were attributed to the C–O bond and oxygen species associated with the MSN-NH_2_ framework (Fig. S3C) [22]. The N 1 s spectrum revealed three peaks at 398.9, 399.6, and 401.5 eV, corresponding to amino-N, pyridine-N, and pyrrole-N, respectively (Fig. S3D) [23]. A strong Si 2p peak at 103.1 eV was observed, confirming the presence of Si species in the material (Fig. S3E) [24]. These XPS results systematically revealed the surface elemental composition and chemical states of MSN-NH₂, providing compelling evidence for the successful synthesis and amino modification of the material. In addition, XRD analysis showed no impurity-related diffraction peaks in MSN-NH_2_, indicating high material purity (Fig. S4) [25]. The broad and featureless diffraction pattern further confirmed the amorphous-phase structural characteristics of MSN-NH_2_. Collectively, these structural features established MSN-NH_2_ as a promising carrier for supporting multi-enzyme cascade reactions.

Subsequently, MSN-NH_2_ were employed as the immobilization matrix, and AChE, CHO, and HRP were selected as model enzymes to construct a localized multi-enzyme cascade immobilization system (AChE/CHO/HRP@MSN) via a carbodiimide-mediated covalent coupling strategy using EDC/NHS chemistry. Successful enzyme immobilization was confirmed by performing morphological and compositional analyses. SEM and TEM images revealed the disappearance of the characteristic mesoporous architecture and an increase in the particle size, indicating effective immobilization of the enzymes within the MSN-NH_2_ framework (Fig. 1D and E). EDS elemental mapping further demonstrated the uniform distribution of C, N, O, and Si throughout the AChE/CHO/HRP@MSN structure (Fig. 1F). Moreover, the electrostatic interactions during enzyme assembly were systematically investigated via zeta potential analysis (Fig. 1G). The surface charge of MSN-NH_2_ shifted from a slightly positive value (+1.73 mV) to a markedly negative value (−13.4 mV) after enzyme immobilization. This pronounced change was consistent with the introduction of negatively charged protein surfaces, further corroborating the successful electrostatic binding and immobilization of the enzymes onto the MSN-NH_2_ carrier [26]. FT-IR spectroscopy was employed to investigate the chemical composition and surface functional groups of MSN-NH_2_ and AChE/CHO/HRP@MSN (Fig. 1H). The FT-IR spectrum of MSN-NH_2_ exhibited a prominent absorption band at 1052 cm^− 1^ corresponding to the Si–O–Si asymmetric stretching vibration, confirming the successful formation of the silica framework [27]. Following enzyme immobilization, the FT-IR spectrum of AChE/CHO/HRP@MSN revealed a new characteristic peak at 1651 cm^− 1^, which was assigned to the C=O stretching vibration of the amide I band and indicative of the presence of proteinaceous components [28]. Additionally, a broad absorption band centered at 3424 cm^− 1^, corresponding to the N–H stretching vibration, was observed, suggesting the formation of amide bonds between the surface amino groups of MSN-NH_2_ and carboxyl groups of the enzyme molecules via EDC/NHS-mediated crosslinking [29]. These distinct spectral changes provide compelling evidence of the successful covalent immobilization of AChE, CHO, and HRP onto the MSN-NH_2_ carrier.

### Enzymatic loading capacity and catalytic activity of AChE/CHO/HRP@MSN

**Fig. 2:**
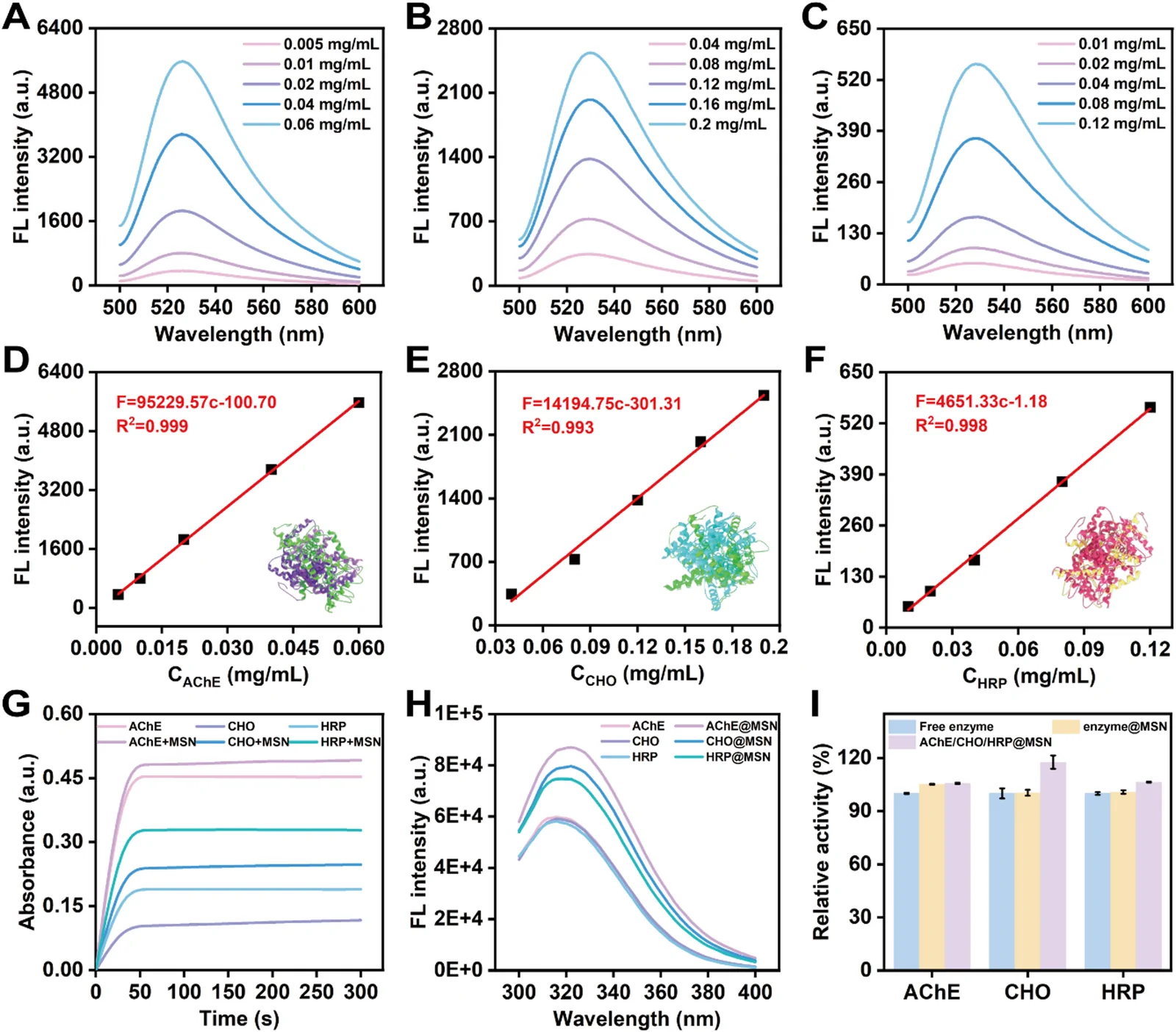
(**A‒C**) Fluorescence curves of FITC-labeled AChE, CHO, and HRP at different concentrations. (**D‒F**) Calibration curves of fluorescence intensity versus the concentration of FITC-labeled AChE, CHO, and HRP. (**G**) Conversion of enzymatic activity after exposure to the MSN-NH_2_ solution and an equal amount of free enzyme as the control. (**H**) Fluorescence spectra of free enzymes and the enzymes immobilized by MSN-NH_2_. (**I**) Conversion of the catalytic activity of free enzymes and immobilized enzymes

A major challenge in immobilization-assisted signal amplification strategies for sensing lies in achieving high enzyme loading while preserving enzymatic catalytic activity. To evaluate the enzyme loading capacity and immobilization efficiency of the multi-enzyme system, standard calibration curves (Fig. 2A‒F) were established to quantify the relative amounts of AChE, CHO, and HRP immobilized on MSN-NH_2_ [30]. The results revealed loading capacities of 0.9%, 4.4%, and 2.6% for AChE, CHO, and HRP, respectively. Notably, significant differences in immobilization efficiency were observed among the three enzymes: AChE exhibited an immobilization efficiency of 26.7%, whereas CHO and HRP achieved considerably higher efficiencies of 80.4% and 88.1%, respectively. These disparities were likely attributable to differences in the enzyme molecular size, surface-charge distribution, and strength of interactions between enzyme molecules and the carrier matrix. The influence of the carrier on enzymatic function is critical for evaluating the feasibility of immobilization [31]. All three enzymes retained, and even exhibited slightly enhanced, catalytic activity after co-incubation with MSN-NH_2_ compared with their free counterparts (Fig. 2G). These results indicate that MSN-NH_2_ exerts negligible inhibitory interference on enzyme activity. The surface properties of the carrier do not appear to disrupt the native catalytic function of the enzymes and may instead provide a local microenvironment conducive to maintaining enzymatic stability and efficient catalysis. Collectively, these findings established a solid foundation for the application of MSN-NH_2_ as a robust and effective enzyme immobilization platform in multi-enzyme cascade biosensing systems.

The conformational integrity of the enzymes before and after immobilization was systematically evaluated using intrinsic fluorescence spectroscopy. Enzymes containing tryptophan residues exhibit a characteristic fluorescence emission peak at approximately 334 nm, which is highly sensitive to changes in the tertiary structure. Therefore, shifts in the emission peak can indicate structural perturbation upon immobilization [32]. No significant shift was observed in the maximum emission wavelengths of immobilized AChE@MSN, CHO@MSN, and HRP@MSN compared with those of their free enzyme counterparts, suggesting that the native tertiary structures of the enzymes are preserved well during the immobilization process (Fig. 2H). To further assess functional retention, a comparative analysis of enzymatic activity was conducted before and after immobilization (Fig. 2I). The results showed excellent activity retention for all three enzymes immobilized on the MSN-NH_2_ carrier, with relative activities reaching 105.18% (AChE), 100.36% (CHO), and 100.78% (HRP) compared with the relative activities of their free forms. Remarkably, when the enzymes were co-immobilized within the MSN-NH_2_ framework, their catalytic activities increased further. This enhancement was likely attributable to the favorable microenvironment provided by the mesoporous matrix and the spatial proximity among enzymes that facilitated substrate channeling and promoted inter-enzyme synergistic interactions.

**Fig. 3:**
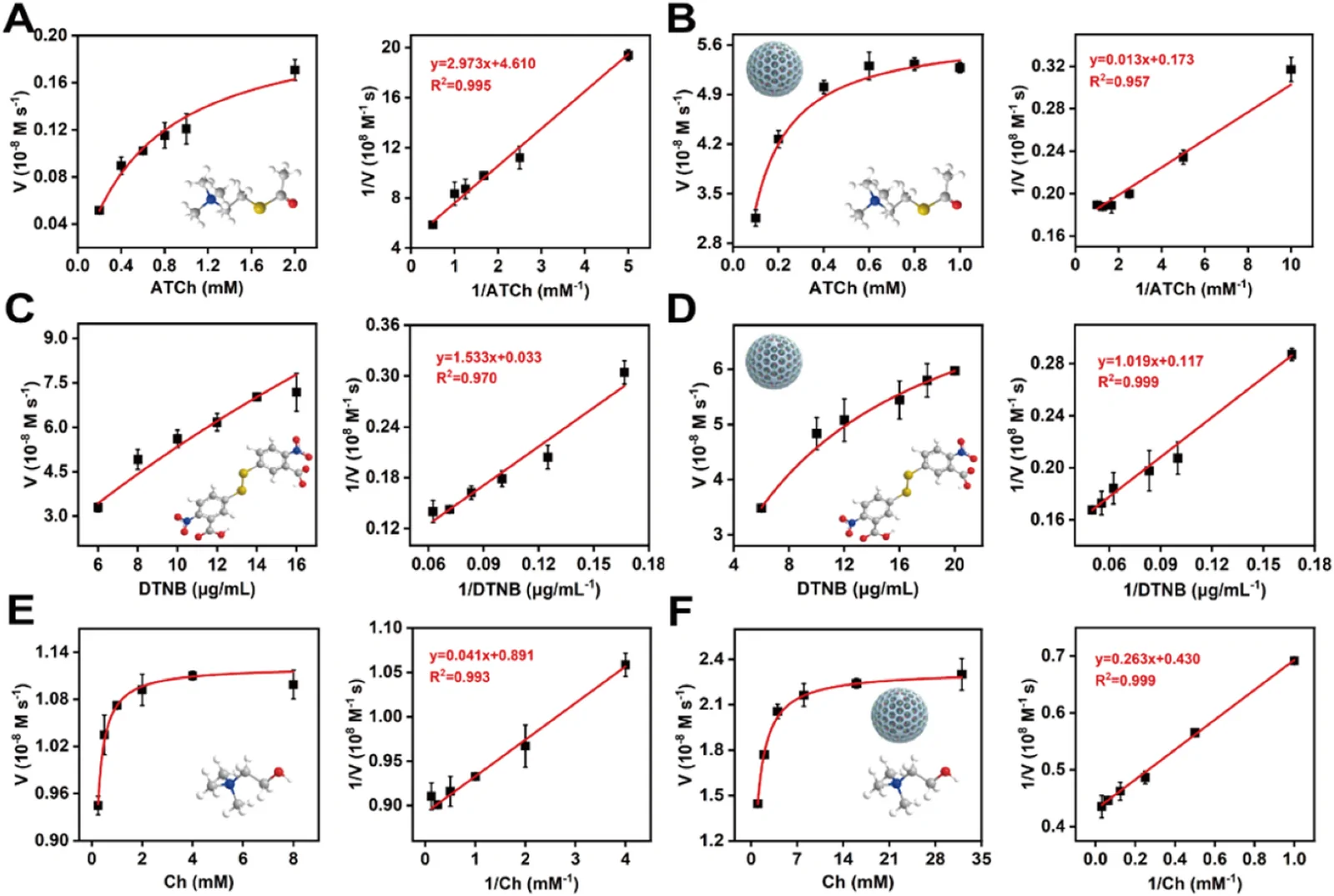
Catalytic kinetic curves of free AChE versus immobilized AChE and free CHO versus immobilized CHO. Steady-state kinetics and corresponding Lineweaver–Burk plots of (**A**) free AChE versus (**B**) immobilized AChE using ATCh as the substrate. Steady-state kinetics and corresponding Lineweaver–Burk plots of (**C**) free AChE versus (**D**) immobilized AChE using DTNB as the substrate. Steady-state kinetics and corresponding Lineweaver–Burk plots of (**E**) free CHO versus (**F**) immobilized CHO using Ch as the substrate

Steady-state kinetic analyses were conducted to systematically evaluate and compare the catalytic performances of immobilized enzymes with those of their free counterparts. Michaelis–Lineweaver–Burk double reciprocal plots and Menten curves were generated by varying the substrate concentrations, and the corresponding kinetic parameters (K_m_ and V_max_) were calculated based on the Lineweaver–Burk Eqs [33, 34]. For AChE, kinetic assays using ATCh as the substrate revealed a substantial decrease in K_m_ from 0.638 (free AChE) to 0.076 mM (immobilized AChE), which was accompanied by an increase in V_max_ from 0.215 × 10^–8^ to 5.791 × 10^–8^ M/s (Fig. 3A and B; Table S1). When DTNB was used as the detection reagent, the K_m_ values for free and immobilized AChE were 9.639 and 8.746 mM, respectively, with corresponding V_max_ values of 7.239 × 10^–8^ and 8.582 × 10^–8^ M/s (Fig. 3C and D; Table S1). When choline served as the substrate, the K_m_ values for free and immobilized CHO were 0.046 and 0.612 mM, respectively, with corresponding V_max_ values of 1.122 × 10^–8^ and 2.325 × 10^–8^ M/s (Fig. 3E and F; Table S2). When TMB was used as the substrate, the K_m_ values for free and immobilized CHO were 0.002 and 0.005 mM and the V_max_ values were 0.851 × 10^–8^ and 1.987 × 10^–8^ M/s, respectively (Fig. S5A and S5B; Table S2). For HRP, kinetic studies using H_2_O_2_ as the substrate showed that the K_m_ increased from 0.175 (free HRP) to 0.349 mM (immobilized HRP) and V_max_ increased from 68.494 × 10^–8^ to 89.841 × 10^–8^ M/s (Fig. S5C and S5D; Table S3). When TMB was used as the substrate, the K_m_ values were 0.441 and 1.802 mM for free and immobilized HRP, respectively, with corresponding V_max_ values of 5.854 × 10^–8^ and 6.282 × 10^–8^ M/s (Fig. S5E and S5F; Table S3). Collectively, these kinetic results indicated that the immobilized enzymes not only retained their catalytic activity but, in several cases, demonstrated enhanced performance. The observed improvements were likely due to the favorable microenvironment and structural confinement provided by the MSN-NH_2_ carrier, which facilitated substrate accessibility and enhanced enzyme stability.

### Feasibility of cascade catalytic sensing of AChE/CHO/HRP@MSN

**Fig. 4:**
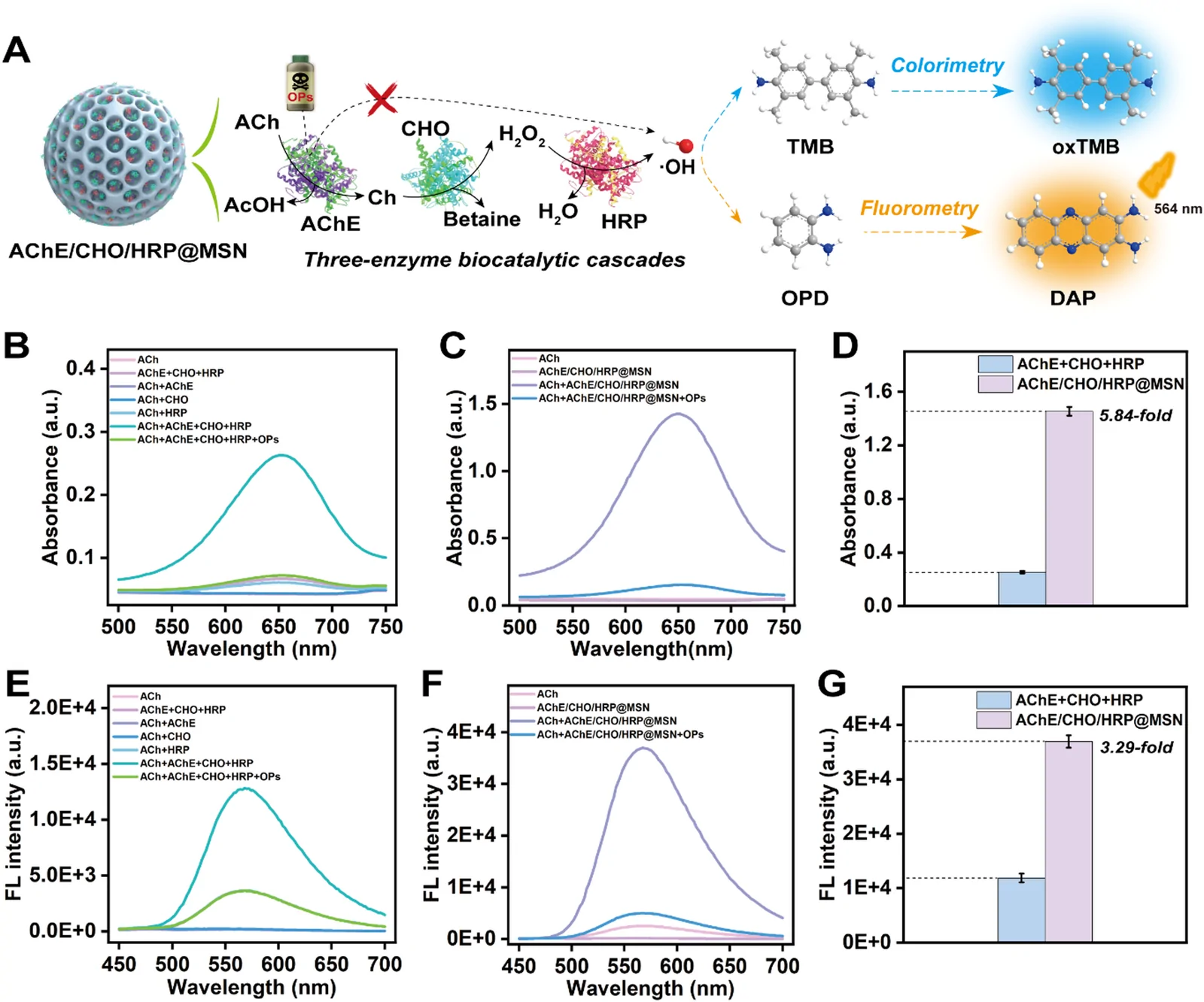
(**A**) Schematic of the sensing mechanism for OPs using the AChE/CHO/HRP@MSN nanoreactor. (**B**) UV–vis absorption spectra of ACh, ACh+AChE, ACh+CHO, ACh+HRP, AChE+CHO+HRP, ACh+AChE+CHO+HRP, and ACh+AChE+CHO+HRP+OPs. (**C**) UV–vis absorption spectra of ACh, AChE/CHO/HRP@MSN, ACh+AChE/CHO/HRP@MSN, and ACh+AChE/CHO/HRP@MSN+OPs. (**D**) Comparison between the catalytic activities of free and immobilized systems in the colorimetric mode. (**E**) Fluorescence spectra of ACh, ACh+AChE, ACh+ CHO, ACh+HRP, AChE+CHO+HRP, ACh+AChE+CHO+HRP, and ACh+AChE+CHO+HRP+OPs. (**F**) Fluorescence spectra of ACh, AChE/CHO/HRP@MSN, ACh+AChE/CHO/HRP@MSN, and ACh+AChE/CHO/HRP@MSN+OPs. (**G**) Comparison between the catalytic activities of free and immobilized systems in the fluorescence mode

The AChE/CHO/HRP@MSN nanoreactor incorporating three cascade enzymes (AChE, CHO, and HRP) catalyzed a sequential multi-step reaction (Fig. 4A). Initially, AChE catalyzed the hydrolysis of ACh to generate Ch. Subsequently, CHO oxidized Ch to produce H_2_O_2_, which then served as the substrate for HRP to generate ·OH. These highly reactive radicals oxidized TMB into its blue-colored oxidized form (oxTMB) or oxidized OPD into a fluorescent compound named 2,3-diaminophenazine (DAP) [29, 35]. In the absence of OPs, AChE retained full catalytic activity, thereby enabling the cascade reaction to proceed and yielding strong colorimetric and fluorescent signals. However, upon OP exposure, AChE activity was significantly inhibited, resulting in decreased H_2_O_2_ generation, subsequent attenuated oxidation of TMB and OPD, and diminished absorbance and fluorescence intensity [36]. The colorimetric response was systematically evaluated to validate the feasibility of this cascade-based sensing mechanism. A significant increase in absorbance was observed only when AChE, CHO, and HRP were present together with ACh (Fig. 4B). In contrast, the addition of OPs led to a marked reduction in absorbance. Control experiments confirmed that individual enzymes, ACh alone, or partial enzyme combinations did not elicite a comparable signal; this result confirmed the necessity of the complete enzymatic cascade for efficient color development and demonstrated the successful construction of the colorimetric sensing platform. The colorimetric performance of the localized multi-enzyme cascade system was further evaluated (Fig. 4C). A pronounced absorbance signal was observed only when both the AChE/CHO/HRP@MSN nanoreactor and ACh were present, whereas the addition of OPs significantly suppressed the signal. Neither the nanoreactor alone nor ACh alone produced a significant absorbance change, confirming the specificity and functionality of the AChE/CHO/HRP@MSN system for colorimetric detection of OPs. Notably, the immobilization system exhibited a 5.84-fold increase in cascade catalytic efficiency compared with the free enzyme mixture (Fig. 4D), highlighting the remarkable advantage of the immobilization strategy in enhancing catalytic performance.

Subsequently, the fluorescence-based detection capabilities of the system were investigated. A significant fluorescence signal was detected only when AChE, CHO, and HRP were present simultaneously with ACh, while the presence of OPs resulted in a substantial decrease in fluorescence intensity (Fig. 4E). Negligible fluorescence changes occurred when only individual components or partial combinations were present, further confirming the successful construction of the fluorescence-based cascade reaction system. The functional performance of AChE/CHO/HRP@MSN in the fluorescence modality was further assessed (Fig. 4F), showing strong fluorescence emission only in the presence of both AChE/CHO/HRP@MSN and ACh, while the introduction of OPs caused a sharp decline in signal. The immobilization system exhibited a significantly enhanced fluorescence response, which was a 3.29-fold increase in cascade catalytic efficiency relative to that of the free enzyme mixture (Fig. 4G). This enhancement was attributed to the proximity effect within the confined nanospaces of the MSN-NH_2_, which shortens the diffusion distances of intermediates, minimizes their loss, and facilitates more efficient sequential reactions. Together, these findings highlight the effectiveness of the multi-enzyme cascade system for the colorimetric and fluorescent dual-mode biosensor, providing a robust platform for highly sensitive OP detection and a foundation for the development of next-generation biosensors.

In addition to catalytic performance, the environmental and storage stability of the AChE/CHO/HRP@MSN nanoreactor was systematically evaluated as a key indicator of its practical sensing applicability [37]. The nanoreactor exhibited high resistance to harsh conditions, including variations in the temperature and pH and exposure to a wide range of organic solvents such as ethanol, ether, and acetone (Fig. S6A‒E). When stored at 4 °C, AChE/CHO/HRP@MSN retained over 95% of its initial activity after 3 days, whereas the free enzyme solution retained only 74.5%. Even after 7 days, the nanoreactor preserved 80.67% of its activity, whereas the free enzyme lost nearly 50%. This enhanced stability was attributed to the high surface area and spatial confinement effect of MSN-NH_2_, which preserves the structural integrity of immobilized enzymes. These results demonstrate that MSN-NH_2_ not only facilitates high enzyme activity and stability but also endows the immobilized system with excellent long-term storage potential, supporting its practical use in biosensing applications.

### Colorimetric and fluorescence dual-mode biosensing for OPs

**Fig. 5:**
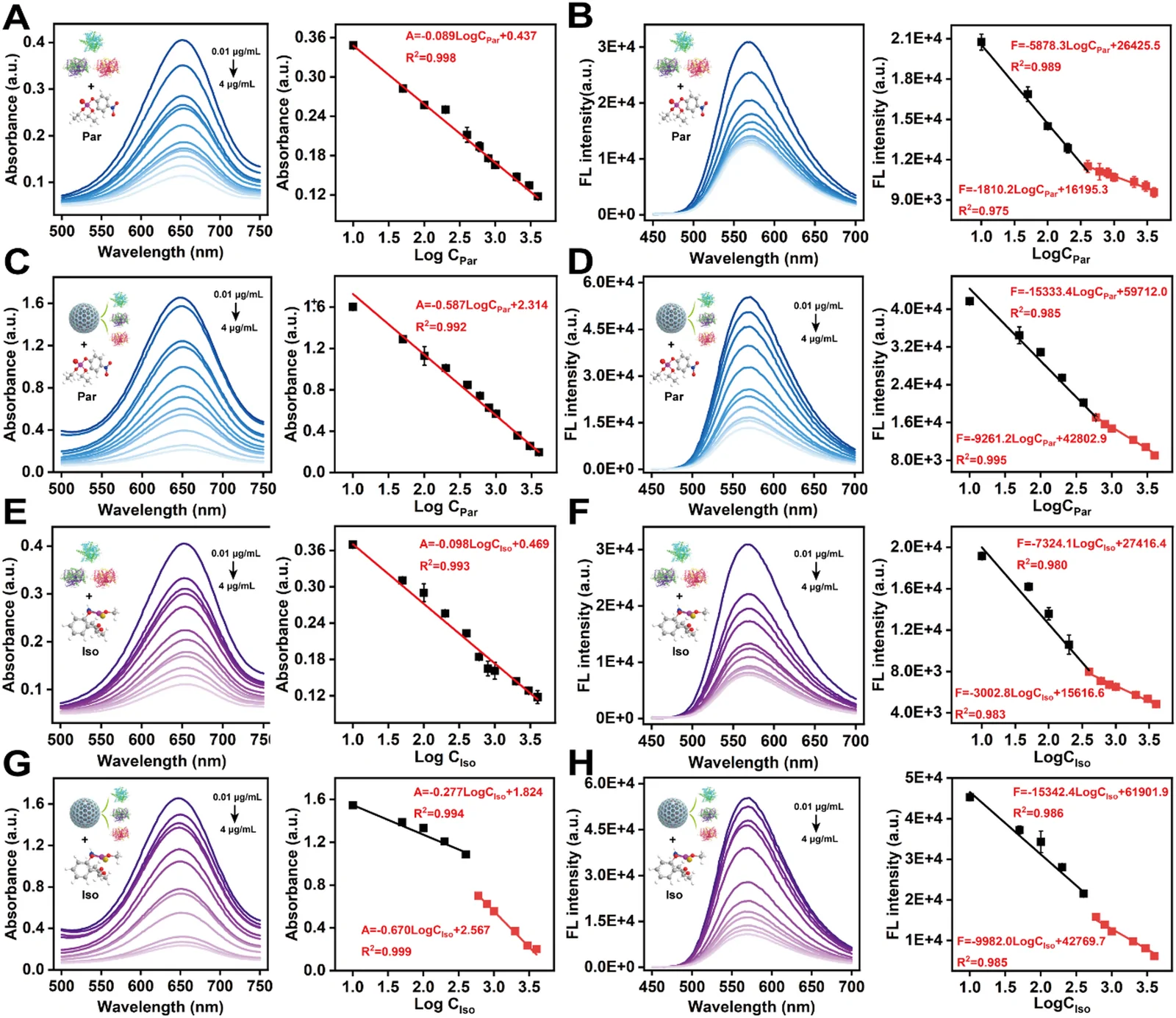
(**A**) UV–vis absorption spectra of the AChE+CHO+HRP system after incubation with different concentrations of paraoxon and corresponding linear plots. (**B**) Fluorescence spectra of the AChE+CHO+HRP system after incubation with different concentrations of paraoxon and corresponding linear plots. (**C**) UV–vis absorption spectra of the AChE/CHO/HRP@MSN system after incubation with different concentrations of paraoxon and corresponding linear plots. (**D**) Fluorescence spectra of the AChE/CHO/HRP@MSN system after incubation with different concentrations of paraoxon and corresponding linear plots. (**E**) UV–vis absorption spectra of the AChE+CHO+HRP system after incubation with different concentrations of isocarbophos and corresponding linear plots. (**F**) Fluorescence spectra of the AChE+CHO+HRP system after incubation with different concentrations of isocarbophos and corresponding linear plots. (**G**) UV–vis absorption spectra of the AChE/CHO/HRP@MSN system after incubation with different concentrations of isocarbophos and corresponding linear plots. (**H**) Fluorescence spectra of the AChE/CHO/HRP@MSN system after incubation with different concentrations of isocarbophos and corresponding linear plots

To maximize catalytic efficiency, key reaction parameters including the reaction temperature, HAc-NaAc buffer pH, concentrations of ACh, TMB, and OPD, as well as reaction and incubation times were systematically optimized (Fig. S7‒S12). The optimal conditions were identified as follows: 37 °C, pH = 7.0, 0.8 mM ACh, 0.2 mM TMB, 4 mM OPD, with TMB/OPD reaction times of 20/40 min, respectively. ACh and OPs were incubated for 40 min, and 1 min, respectively. Under these optimized conditions, the biosensing system exhibited maximum signal response. Five representative OPs, dichlorvos, dimethoate, phosalone, paraoxon, and isocarbophos, were selected as analytes. As OPs inhibited AChE activity, they suppressed the cascade generation of H_2_O_2_ and ·OH, leading to decreased oxidation of TMB (colorimetric) and OPD (fluorescent), and consequently reduced their signal intensities. Under optimized conditions, colorimetric and fluorescence dual-signal responses (absorbance at 652 nm and fluorescence intensity at 564 nm) were measured over an OP concentration range of 10–4000 ng/mL. The detection performance of both the free and immobilized systems was systematically evaluated using paraoxon and isocarbophos as representative analytes. The absorbance at 652 nm and fluorescence intensity at 564 nm of both systems decreased (Fig. 5A‒D) with increasing paraoxon concentrations (10–4000 ng/mL), exhibiting excellent linear correlations with the logarithmic concentration of paraoxon (R^2^ > 0.98). Based on the 3σ/s criterion, the calculated limit of detection (LOD) of paraoxon in the colorimetric mode was significantly reduced from 8.983 ng/mL (free system) to 1.050 ng/mL (immobilized system). Similarly, in the fluorescence mode, the LOD decreases from 3.671 to 1.288 ng/mL. Consistent results were obtained for isocarbophos (Fig. 5E‒H), with both systems demonstrating strong linear responses (R^2^ > 0.98) across the same concentration range (10–4000 ng/mL). The LODs of the immobilized system reached 1.110 (colorimetric) and 1.288 ng/mL (fluorescent), in contrast to 7.343 and 2.840 ng/mL for the free system, respectively [38]. Compared with the free system, the immobilized platform enhanced detection sensitivity by 8.55-fold and 6.62-fold for paraoxon and isocarbophos, respectively, in the colorimetric mode, and by 2.85-fold and 2.20-fold in the fluorescence mode. Collectively, these results highlight the substantial advantages of the immobilization strategy in enhancing detection sensitivity and overall analytical performance.

The detection performance of three additional OPs (dichlorvos, dimethoate, and phosalone) was further evaluated. Both the absorbance and fluorescence intensity exhibited clear dose-dependent responses over a concentration range of 10–4000 ng/mL (Fig. S13‒S15), with strong linear correlations to the logarithm of analyte concentration (R^2^ > 0.98). The key performance parameters of the sensors are summarized in Table S4 and S5. Compared with the free enzyme system, the immobilized platform demonstrated markedly enhanced sensitivity in both detection modes. In the colorimetric mode, the sensitivity improvements were 7.41-fold, 5.60-fold, and 7.01-fold for dichlorvos, dimethoate, and phosalone, respectively, and those in the fluorescence mode were 2.11-fold, 1.93-fold, and 2.35-fold. This superior performance was primarily attributed to: (1) the preservation of high catalytic activity and stability through enzyme immobilization; (2) the proximity effect in localized cascade catalysis, which minimizes intermediate decomposition and substrate diffusion loss; and (3) the reduced mass-transfer resistance provided by the nanoscale architecture, enabling more efficient enzymatic reactions. Compared with previously reported OP detection strategies (Table S6), the proposed dual-mode biosensor offers a broader dynamic range and substantially improved sensitivity. These advantages stem from the synergistic combination of dual-signal readout, spatially confined multi-enzyme catalysis, and nanostructure-driven stabilization, collectively establishing a robust platform for next-generation biosensor development.

Specificity and anti-interference capabilities are critical parameters for evaluating biosensor performance [39, 40]. To assess the specificity of the AChE/CHO/HRP@MSN sensor, various non-OPs, including carbendazim, acetamiprid, fipronil, tebuconazole, cypermethrin, deltamethrin, and fenvalerate, were tested (Fig. S16). Only paraoxon elicited significant changes in absorbance and fluorescence intensity, while the other pesticides caused negligible signal variations. These results confirmed the high specificity of the biosensor towards paraoxon. To further validate the anti-interference performance, common biomolecules (BSA, glucose, sucrose, and arginine) and ions (Na⁺, K⁺, and Cl⁻) were introduced into the system (Fig. S17). No significant changes in dual-signal outputs were observed, demonstrating strong resistance to matrix interference and high reliability in complex sample environments. The universality of the biosensor was further confirmed by testing additional OP compounds, including phenthoate, parathion, methyl parathion, methamidophos, acephate, malathion, and glyphosate (Fig. S18). Consistent signal responses across different OPs validated the broad-spectrum applicability of the system. These results collectively demonstrate that the AChE/CHO/HRP@MSN sensing platform possesses excellent selectivity, anti-interference capability, and universality, making it suitable for practical applications in environmental and agricultural sample analysis.

### Smartphone-assisted hydrogel biosensing for on-site OP detection

**Fig. 6:**
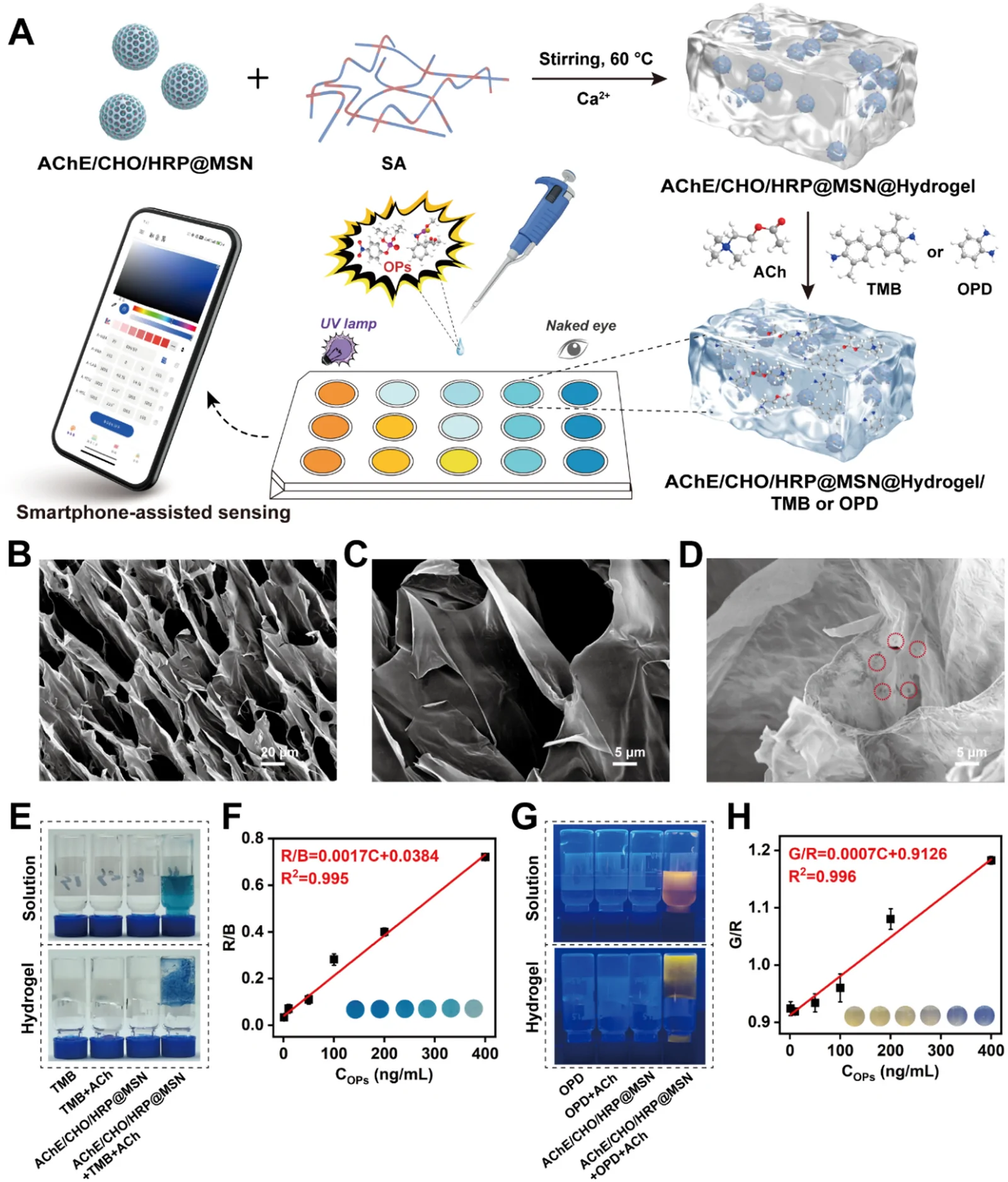
(**A**) Schematic of the smartphone-assisted AChE/CHO/HRP@MSN@Hydrogel biosensor. (**B**,** C**) SEM images of the SA hydrogel. (**D**) SEM image of AChE/CHO/HRP@MSN@Hydrogel. Red circles indicate where the AChE/CHO/HRP@MSN nanoreactors were located inside the hydrogel. (**E**) Colorimetric photographs of the solution and hydrogel systems. (**F**) Linear relationship between the RGB values and paraoxon concentration (1–400 ng/mL) in the colorimetric mode (*n* = 3). (**G**) Fluorescence photographs of the solution and hydrogel systems. (**H**) Linear relationship between the RGB values and paraoxon concentration (1–400 ng/mL) in the fluorescence mode (*n* = 3)

To enable rapid on-site detection of OPs, a smartphone-assisted, portable hydrogel-based biosensing platform with dual colorimetric and fluorescence readouts was developed (Fig. 6A). The system incorporates the AChE/CHO/HRP@MSN nanoreactor uniformly dispersed within a SA hydrogel matrix, enabling highly sensitive OP detection. Upon incubation with OPs for 10 min, followed by the sequential addition of ACh, TMB, and HAc–NaAc buffer, the AChE/CHO/HRP@MSN@Hydrogel displayed concentration-dependent colorimetric and fluorescent responses. Smartphone-based signal acquisition was achieved using the built-in camera and Color Picker application to extract RGB values from hydrogel images in real time. Standard calibration curves correlating the RGB ratios with paraoxon concentrations were constructed for both of these modes. SEM images showed that the SA hydrogel had an interconnected 3D porous structure (Fig. 6B and C), and the AChE/CHO/HRP@MSN nanoreactor was uniformly distributed inside the porous hydrogel (Fig. 6D). This structural feature ensured the uniformity and stability of the color signal measurement. In the colorimetric mode, a distinct blue shift was visible to the naked eye (Fig. 6E), and the R/B ratio exhibited a good linear relationship with paraoxon concentration (1–400 ng/mL), with the regression equation R/B = 0.0017 C + 0.0384 (R^2^ = 0.995) (Fig. 6F). Similarly, a significant color change of the hydrogel was observed in the fluorescence mode (Fig. 6G), and the G/R ratio showed strong linearity with paraoxon concentration (1–400 ng/mL), with a regression equation of G/R = 0.0007 C + 0.9126 (R^2^ = 0.996) (Fig. 6H). These results confirm that this portable hydrogel-based dual-mode biosensor enables rapid, sensitive, and visualized detection of OPs, offering a reliable, low-cost platform for real-time environmental and food monitoring using readily available mobile devices.

### Application in actual samples

To validate the practical applicability of the dual-mode AChE/CHO/HRP@MSN biosensor, OP residue detection was performed in real samples, including tap water, cabbage, and apples, using the standard addition method. Paraoxon was spiked at concentrations of 50, 100, and 200 ng/mL to assess detection accuracy in complex matrices. The recoveries in the colorimetric and fluorescence modes ranged from 97.53% to 114.06% and 95.26% to 108.22%, respectively, confirming the high accuracy of the sensor in real sample analysis (Table S7). Furthermore, the relative standard deviations (RSDs) of the colorimetric and fluorescence methods were lower than 8.51% and 6.99%, respectively, confirming excellent reproducibility. To further assess the inter-batch reproducibility of the biosensors, seven independent batches of the AChE/CHO/HRP@MSN material were prepared and tested (Fig. S19). All batches produced consistent absorbance and fluorescence responses, with RSDs below 5%, validating the robustness and stability of the fabrication process. These results fully confirm that the dual-mode biosensor is capable of real-time visualization and monitoring of residual OPs in complex real samples with good practicality.

**Fig. 7:**
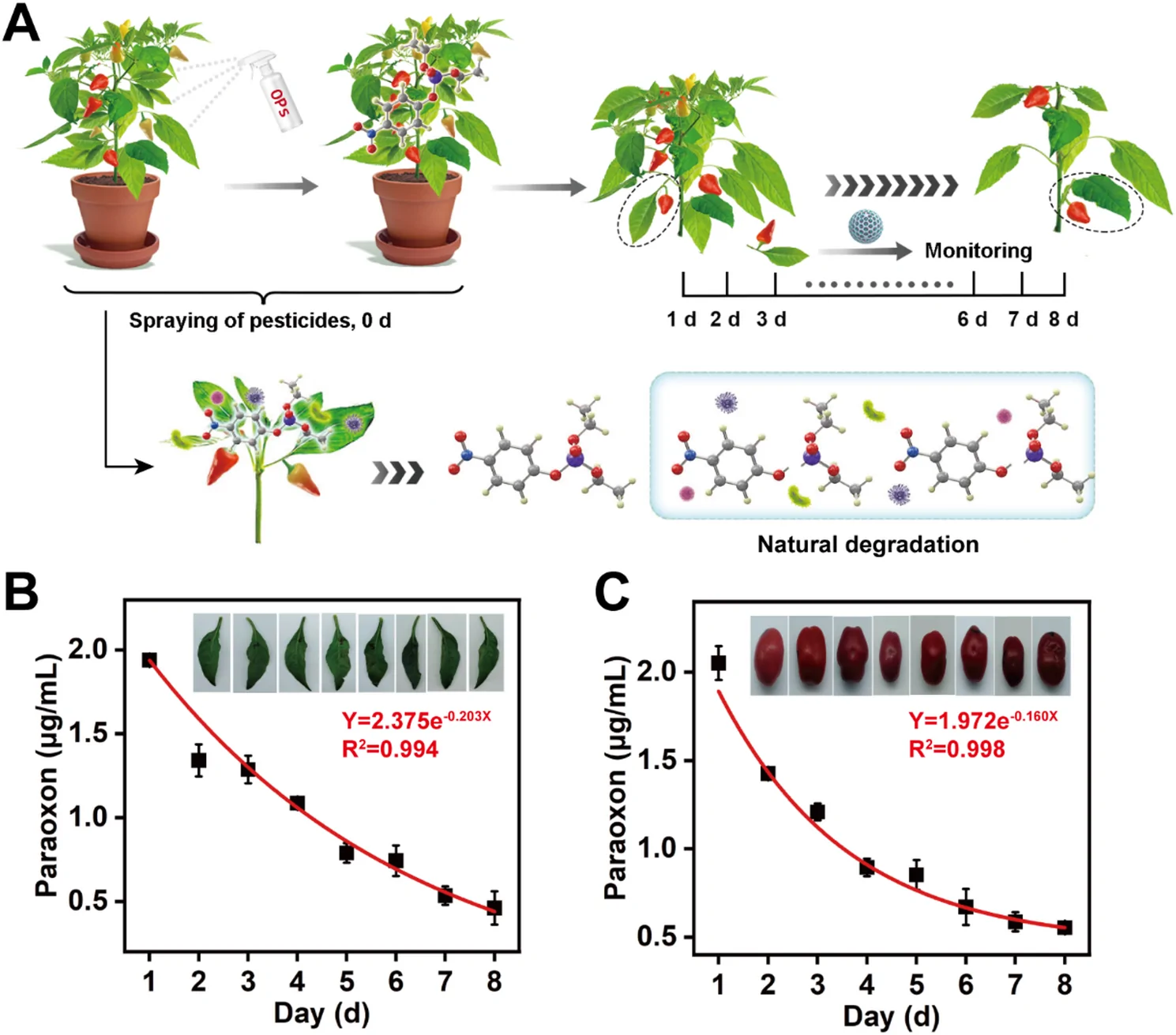
(**A**) Schematic of the AChE/CHO/HRP@MSN biosensor for monitoring the degradation of OPs in peppers. (**B**) Degradation curve of paraoxon in pepper leaves. (**C**) Degradation curve of paraoxon in pepper fruits

Monitoring the degradation behavior of pesticides is essential for evaluating their environmental impact, especially in mitigating exposure risks, controlling pollution, and optimizing the degradation process [41]. In this study, the AChE/CHO/HRP@MSN biosensor was employed for in situ, real-time monitoring of paraoxon degradation in pepper samples. Paraoxon was sprayed onto pepper surfaces to simulate actual pesticide application, and the biosensor was used to dynamically monitor the degradation process of paraoxon in pepper leaves and fruits over an 8-day growing period to assess its environmental behavior (Fig. 7A). The degradation kinetics followed a pseudo-first-order model, with rate equations of Y = 2.375 e^–0.203X^ (R^2^ = 0.994) for the leaves (Fig. 7B) and Y = 1.972 e^–0.160X^ (R^2^ = 0.998) for the fruits (Fig. 7C). These results highlight the robust capability of the AChE/CHO/HRP@MSN biosensor for dynamic, real-time monitoring of pesticide dissipation, offering a powerful and practical platform for environmental risk assessment, pollutant tracking, and sustainable agrochemical management.

## Conclusions

We successfully synthesized MSN-NH_2_ having large specific surface areas and multi-porosity and employed them as an efficient carrier to immobilize AChE, CHO, and HRP in situ, forming a spatially confined multi-enzyme cascade catalytic system. The prepared AChE/CHO/HRP@MSN nanoreactor exhibited superior enzymatic activity and stability compared with its free enzyme counterparts. The spatial confinement of enzymes within the MSN matrix effectively shortened the diffusion distances of reactants and intermediates, thereby enhancing substrate channeling and catalytic efficiency and significantly improving the sensitivity of OP detection. By further integrating AChE/CHO/HRP@MSN into a smartphone-assisted hydrogel platform, we developed a portable, low-cost, and visual biosensor for rapid on-site OP monitoring. Notably, the system enabled real-time tracking of OP degradation in peppers, highlighting its potential for long-term environmental surveillance and food safety assessment. Despite the system’s advantages, several limitations remain: (1) the assay time requires further reduction to improve field applicability, (2) the current system provides broad-spectrum OP detection but lacks specificity toward structurally similar OP analogs, and (3) the platform remains at the proof-of-concept stage and requires further optimization for commercialized deployment. Future work should focus on the integration of chemical fingerprinting strategies to enhance selectivity and system scaling for real-world application scenarios.

## Supplementary Information

Supplementary Material 1

## Data Availability

No datasets were generated or analysed during the current study.
