# Supplementary material for: Spatially confined multi-enzyme cascade nanoreactor for portable and real-time pesticide detection
Source: J Nanobiotechnology. 2026 Jun 11;24:778. doi: 10.1186/s12951-026-04677-8 (PMC13488018; doi:10.1186/s12951-026-04677-8)
Supplement: Supplementary file 1 [file 12951_2026_4677_MOESM1_ESM.doc]

**Supplementary Material**

**Spatially confined multi-enzyme cascade nanoreactor for portable and real-time pesticide detection**

Wei Zhang^a,1^, Jia Shu^b,1^, Chonghai Gong^a^, Song Zhou^a^, Hanting Wang^a^, Kunyang Feng^a^, Dongmei Liu^c^, Rui Chen^d^, Juan Li^d,^*, Zhengwei Zhang^c,^*, Siqiao Li^a,^*

^a^ School of Basic Medical Sciences, Chongqing Medical University, Chongqing 400016, China

^b^ Ophthalmology Medical Center, The First Affiliated Hospital of Chongqing Medical University, Chongqing Key Laboratory for the Prevention and Treatment of Major Blinding Eye Diseases, Chongqing Branch (Municipality Division) of National Clinical Research Centre for Ocular Diseases, Chongqing 400016, China.

^c^ College of Pharmacy, Chongqing Medical University, Chongqing 400016, China

^d^ Key Laboratory of Clinical Laboratory Diagnostics (Ministry of Education), College of Laboratory Medicine, Chongqing Medical University, Chongqing 400016, China

*Corresponding author:

E-mail address: juanli0905@163.com (J. Li); zzwcpu@cqmu.edu.cn (Z. Zhang); [lsqzzw@yeah.net](mailto:lsqzzw@yeah.net) (S. Li).

^1^ These authors contributed equally to this work.

**Fig. S1.** HAADF-STEM and corresponding elemental mapping images of MSN-NH_2_.

**Fig. S2.** Pore width distribution curve of MSN-NH_2_.

**Fig. S3.** (A) XPS survey spectra of MSN-NH_2_. (B‒E) High-resolution XPS spectra of C 1s, O 1s, N 1s, and Si 2p for MSN-NH_2_.

**Fig. S4.** XRD pattern of MSN-NH_2_.

**Fig. S5.** Catalytic kinetic curves of free CHO versus immobilized CHO and free HRP versus immobilized HRP. Steady-state kinetics and corresponding Lineweaver–Burk plots of (A) free CHO versus (B) immobilized CHO using TMB as the substrate. Steady-state kinetics and corresponding Lineweaver-Burk plots of (C) free HRP versus (D) immobilized HRP using H_2_O_2_ as substrate. Steady-state kinetics and corresponding Lineweaver-Burk plots of (E) free HRP versus (F) immobilized HRP using TMB as substrate.

**Fig. S6.** (A) Stability of free enzymes and AChE/CHO/HRP@MSN when exposed to different pH HAc-NaAc buffer solutions. (B) Stability of free enzymes and AChE/CHO/HRP@MSN when exposed to different pH PBS buffer solutions. (C) Stability of free enzymes and AChE/CHO/HRP@MSN when exposed to different temperatures. (D) Stability of free enzymes and AChE/CHO/HRP@MSN when exposed to different organic solvents. (E) Stability of free enzymes and AChE/CHO/HRP@MSN after storage.

**Fig. S7.** Optimization of sensing condition: temperature (15, 25, 37, 40, 50, and 60 ℃).

**Fig. S8.** Optimization of sensing condition: incubation time of OPs (1, 2, 4, 6, 8, and 10 min).

**Fig. S9.** Optimization of sensing condition: HAc-NaAc buffer (pH 3, 4, 5, 6, 7, 8, and 9).

**Fig. S10.** Optimize the detection conditions: (A) concentration of ACh (0.08, 0.16, 0.40, 0.80, 1.20, 1.60, and 2.00 mM), and (B) reaction time of ACh (10, 20, 30, 40, 50, 60, and 70 min).

**Fig. S11**. Optimize the detection conditions: (A) concentration of TMB (0.08, 0.12, 0.16, 0.20, 0.40, 0.80, and 1.20 mM), and (B) reaction time of TMB (5, 10, 15, 20, 25, 30, and 35 min).

**Fig. S12.** Optimize the detection conditions: (A) concentration of OPD (1, 2, 4, 8, 12, 16, and 20 mM), and (B) reaction time of OPD (10, 20, 30, 40, 50, 60, and 70 min).

**Fig. S13.** (A) UV-vis absorption spectra of the free enzyme system (AChE+CHO+HRP) following incubation with different concentrations of dichlorvos (0.01–4 μg/mL) and corresponding linear plots. (B) Fluorescence spectra of the free enzyme system (AChE+CHO+HRP) following incubation with different concentrations of dichlorvos (0.01–4 μg/mL) and corresponding linear plots. (C) UV-vis absorption spectra of the AChE/CHO/HRP@MSN biosensing system following incubation with different concentrations of dichlorvos (0.01–4 μg/mL) and corresponding linear plots. (D) Fluorescence spectra of the AChE/CHO/HRP@MSN biosensing system following incubation with different concentrations of dichlorvos (0.01–4 μg/mL) and corresponding linear plots.

**Fig. S14.** (A) UV-vis absorption spectra of the free enzyme system (AChE+CHO+HRP) following incubation with different concentrations of dimethoate (0.01–4 μg/mL) and corresponding linear plots. (B) Fluorescence spectra of the free enzyme system (AChE+CHO+HRP) following incubation with different concentrations of dimethoate (0.01–4 μg/mL) and corresponding linear plots. (C) UV-vis absorption spectra of the AChE/CHO/HRP@MSN biosensing system following incubation with different concentrations of dimethoate (0.01–4 μg/mL) and corresponding linear plots. (D) Fluorescence spectra of the AChE/CHO/HRP@MSN biosensing system following incubation with different concentrations of dimethoate (0.01–4 μg/mL) and corresponding linear plots.

**Fig. S15.** (A) UV-vis absorption spectra of the free enzyme system (AChE+CHO+HRP) following incubation with different concentrations of phosalone (0.01–4 μg/mL) and corresponding linear plots. (B) Fluorescence spectra of the free enzyme system (AChE+CHO+HRP) following incubation with different concentrations of phosalone (0.01–4 μg/mL) and corresponding linear plots. (C) UV-vis absorption spectra of the AChE/CHO/HRP@MSN biosensing system following incubation with different concentrations of phosalone (0.01–4 μg/mL) and corresponding linear plots. (D) Fluorescence spectra of the AChE/CHO/HRP@MSN biosensing system following incubation with different concentrations of phosalone (0.01–4 μg/mL) and corresponding linear plots.

**Fig. S16.** Selectivity of AChE/CHO/HRP@MSN biosensor towards different substances.

**Fig. S17.** Anti-interference performance of AChE/CHO/HRP@MSN biosensor against different interferents.

**Fig. S18.** Universality of AChE/CHO/HRP@MSN biosensor for the detection of various OPs.

**Fig. S19.** Uniformity of AChE/CHO/HRP@MSN from different synthesis batches for OPs sensing.

**Table S1.** Comparison of K_m_ and V_max_ with free AChE and immobilized AChE.

|  | ATCh | | DTNB | |
| --- | --- | --- | --- | --- |
|  | K_m_  (mM) | V_max_  (10^–8^ M/s) | K_m_  (mM) | V_max_  (10^–8^ M/s) |
| Free AChE | 0.638 | 0.215 | 9.636 | 7.239 |
| Immobilized AChE | 0.076 | 5.791 | 8.746 | 8.582 |

**Table S2.** Comparison of K_m_ and V_max_ with free CHO and immobilized CHO.

|  | Ch | | TMB | |
| --- | --- | --- | --- | --- |
|  | K_m_  (mM) | V_max_  (10^–8^ M/s) | K_m_  (mM) | V_max_  (10^–8^ M/s) |
| Free CHO | 0.046 | 1.122 | 0.002 | 0.851 |
| Immobilized CHO | 0.612 | 2.325 | 0.005 | 1.987 |

**Table S3.** Comparison of K_m_ and V_max_ with free HRP and immobilized HRP.

|  | H_2_O_2_ | | TMB | |
| --- | --- | --- | --- | --- |
|  | K_m_  (mM) | V_max_  (10^–8^ M/s) | K_m_  (mM) | V_max_  (10^–8^ M/s) |
| Free HRP | 0.175 | 68.494 | 0.441 | 5.854 |
| Immobilized HRP | 0.349 | 89.841 | 1.802 | 6.282 |

**Table S4.** Main parameters of the colorimetric detection.

| Target | Biosensor | Linear equation | Linear range (ng/mL) | LOD (ng/mL) |
| --- | --- | --- | --- | --- |
| Paraoxon | AChE + CHO + HRP | A = −0.089 LogC_Par_ + 0.437 (R^2^ = 0.998) | 10–4000 | 8.983 |
|  | AChE/CHO/HRP@MSN | A = −0.587 LogC_Par_ + 2.314 (R^2^ = 0.992) | 10–4000 | 1.050 |
| Isocarbophos | AChE + CHO + HRP | A = −0.098 LogC_Iso_ + 0.469 (R^2^ = 0.993) | 10–4000 | 7.343 |
|  | AChE/CHO/HRP@MSN | A = −0.277 LogC_Iso_ + 1.824 (R^2^ = 0.994);  A = −0.670 LogC_Iso_ + 2.567 (R^2^ = 0.999) | 10–400;  600–4000 | 1.110 |
| Dichlorvos | AChE + CHO + HRP | A = −0.092 LogC_DDV_ + 0.451 (R^2^ = 0.999) | 10–4000 | 8.362 |
|  | AChE/CHO/HRP@MSN | A = −0.239 LogC_DDV_ + 1.956 (R^2^ = 0.988);  A = −0.959 LogC_DDV_ + 3.605 (R^2^ = 0.999) | 10–400;  400–4000 | 1.128 |
| Dimethoate | AChE + CHO + HRP | A = −0.103 LogC_Dim_ + 0.429 (R^2^ = 0.996);  A = −0.055 LogC_Dim_ + 0.293 (R^2^ = 0.999) | 10–600;  600–4000 | 6.665 |
|  | AChE/CHO/HRP@MSN | A = −0.166 LogC_Dim_ + 1.817 (R^2^ = 0.999);  A = −0.824 LogC_Dim_ + 3.214 (R^2^ = 0.999) | 10–400;  600–4000 | 1.190 |
| Phosalone | AChE + CHO + HRP | A = −0.088 LogC_Pho_ + 0.388 (R^2^ = 0.990);  A = −0.043 LogC_Pho_ + 0.252 (R^2^ = 0.999) | 10–600;  600–4000 | 9.210 |
|  | AChE/CHO/HRP@MSN | A = −0.106 LogC_Pho_ + 1.723 (R^2^ = 0.994);  A = −0.805 LogC_Pho_ + 3.083 (R^2^ = 0.981) | 10–400;  600–4000 | 1.313 |

**Table S5.** Main parameters of the fluorescence detection.

| Target | Biosensor | Linear equation | Linear range (ng/mL) | LOD (ng/mL) |
| --- | --- | --- | --- | --- |
| Paraoxon | AChE+CHO+HRP | F = −5878.3 LogC_Par_ + 26425.5 (R^2^ = 0.989);  F = −1810.2 LogC_Par_ + 16195.3 (R^2^ = 0.975) | 10–400;  400–4000 | 3.671 |
|  | AChE/CHO/HRP@MSN | F = −15333.4 LogC_Par_ + 59712.0 (R^2^ = 0.985);  F = −9261.2 LogC_Par_ + 42802.9 (R^2^ = 0.995) | 10–600;  600–4000 | 1.288 |
| Isocarbophos | AChE+CHO+HRP | F = −7324.1 LogC_Iso_ + 27416.4 (R^2^ = 0.980);  F = −3002.8 LogC_Iso_ + 15616.6 (R^2^ = 0.983) | 10–400;  400–4000 | 2.840 |
|  | AChE/CHO/HRP@MSN | F = −15342.4 LogC_Iso_ + 61901.9 (R^2^ = 0.986);  F = −9982.0 LogC_Iso_ + 42769.7 (R^2^ = 0.985) | 10–400;  600–4000 | 1.288 |
| Dichlorvos | AChE+CHO+HRP | F= −6191.6 LogC_DDV_ + 29547.0 (R^2^ = 0.990);  F= −3356.5 LogC_DDV_ + 20112.6 (R^2^ = 0.985) | 10–400;  600–4000 | 3.437 |
|  | AChE/CHO/HRP@MSN | F = −7950.9 LogC_DDV_ + 50714.3 (R^2^ = 0.996);  F = −12681.3 LogC_DDV_ + 53175.3 (R^2^ = 0.999) | 10–400;  600–4000 | 1.630 |
| Dimethoate | AChE+CHO+HRP | F = −6025.6 LogC_Dim_ + 31087.4 (R^2^ = 0.995) | 10–4000 | 3.556 |
|  | AChE/CHO/HRP@MSN | F = −6363.3 LogC_Dim_ + 56128.7 (R^2^ = 0.985);  F = −19569.5 LogC_Dim_ + 80614.0 (R^2^ = 0.999) | 10–400;  600–4000 | 1.841 |
| Phosalone | A AChE+CHO+HRP | F = −5928.3 LogC_Pho_ + 27705.5 (R^2^ = 0.982);  F = −1825.2 LogC_Pho_ + 16521.3 (R^2^ = 0.997) | 10–400;  400–4000 | 3.631 |
|  | AChE/CHO/HRP@MSN | F = −8945.4 LogC_Pho_ + 58316.5 (R^2^ = 0.976);  F = −13842.7 LogC_Pho_ + 57231.7 (R^2^ = 0.998) | 10–400;  600–4000 | 1.544 |

**Table S6.** Comparison of different biosensors for the detection of OPs.

| Detection  sensor | Detection  method | Linear  range | LOD | References |
| --- | --- | --- | --- | --- |
| NH_2_-CuBDC | Colorimetry | 1.8–180 ng/mL | 1.57 ng/mL | (Liu et al., 2024) |
|  | Fluorometry | 4.5–450 ng/mL | 2.33 ng/mL |  |
| GSH-Fe | Colorimetry | 0.3–10 μg/mL | 0.3 μg/mL | (Yan et al., 2024) |
| W/LC | Colorimetry | 1–10000 ng/mL | 1.9 ng/mL | (B. Li et al., 2024) |
| AuNCs@MnO_2_ | Fluorometry | 5.0–500 ng/mL | 5.0 ng/mL | (H. Li et al., 2022) |
| Zr/F/MS | Fluorometry | 50–2500 μg/L | 4.95 μg/L | (Zhang et al., 2022) |
| Au NBPs@Fe-MOF | Colorimetry | 0.01–0.8 μg/mL | 0.01 μg/mL | (Wang et al., 2023) |
| Pt@ZIF-8@TMS | Colorimetry | 0–500 ng/mL | 0.7 ng/mL | (Shen et al., 2025) |
| PtCu@HRP@ZIF-8 | Fluorometry | 6–800 ng/mL | 1.8 ng/mL | (Huang et al., 2022) |
| AChE-MnO_2_@HPH | Colorimetry | 4–400 ng/mL | 0.63 ng/mL | (Zhu et al., 2022) |
| AChE@Zn-MOF-74 | Colorimetry | 1.0–600 ng/mL | 1.0 ng/mL | (H. Li et al., 2024) |
| AChE/CHO/HRP@MSN | Colorimetry | 10–4000 ng/mL | 1.050 ng/mL | This work |
|  | Fluorometry | 10–600 ng/mL;  600–4000 ng/mL | 1.288 ng/mL |  |

**Table S7.** Practical feasibility of the AChE/CHO/HRP@MSN sensing platform for OPs detection.

| Samples | Spiked  (ng/mL) | Colorimetric | | | Fluorescent | | |
| --- | --- | --- | --- | --- | --- | --- | --- |
|  |  | Found  (ng/mL) | Recovery  (%) | RSD  (%, n=3) | Found  (ng/mL) | Recovery  (%) | RSD  (%, n=3) |
| Cabbage | 0 | ND |  |  | ND |  |  |
|  | 50 | 57.03 | 114.06 | 4.05 | 50.36 | 100.72 | 3.08 |
|  | 100 | 106.23 | 106.23 | 7.72 | 98.14 | 98.14 | 6.08 |
|  | 200 | 199.45 | 99.73 | 7.27 | 208.42 | 104.21 | 4.22 |
| Apple | 0 | ND |  |  | ND |  |  |
|  | 50 | 48.80 | 97.59 | 3.20 | 47.63 | 95.26 | 1.80 |
|  | 100 | 101.67 | 101.67 | 4.05 | 99.64 | 99.64 | 6.31 |
|  | 200 | 197.49 | 98.74 | 2.85 | 195.48 | 97.74 | 1.49 |
| Tap water | 0 | ND |  |  | ND |  |  |
|  | 50 | 49.34 | 98.68 | 3.21 | 49.57 | 99.14 | 6.99 |
|  | 100 | 97.53 | 97.53 | 8.51 | 108.22 | 108.22 | 1.59 |
|  | 200 | 204.97 | 102.48 | 7.82 | 209.02 | 104.51 | 4.12 |

**References**

Huang, J., Jiao, L., Xu, W., Wang, H., Sha, M., Wu, Z., Gu, W., Hu, L., & Zhu, C. (2022). Amorphous metal-organic frameworks on PtCu hydrogels: Enzyme immobilization platform with boosted activity and stability for sensitive biosensing. *Journal of Hazardous Materials*, *432*, 128707. https://doi.org/10.1016/j.jhazmat.2022.128707

Li, B., Wu, W., Lin, J.-M., Wang, T., Hu, Q., & Yu, L. (2024). Water in liquid crystal emulsion-based sensing platform for colorimetric detection of organophosphorus pesticide. *Food Chemistry*, *436*, 137732. https://doi.org/10.1016/j.foodchem.2023.137732

Li, H., Lu, Q., Shi, J., Zhang, X., Sun, P., Yan, X., & Lu, G. (2024). Enzyme‐Engineered Metal‐Organic Frameworks for the Construction of Organophosphorus Pesticide Biosensor. *Advanced Functional Materials*, *34*(6), 2309383. https://doi.org/10.1002/adfm.202309383

Li, H., Zou, R., Su, C., Zhang, N., Wang, Q., Zhang, Y., Zhang, T., Sun, C., & Yan, X. (2022). Ratiometric fluorescent hydrogel for point-of-care monitoring of organophosphorus pesticide degradation. *Journal of Hazardous Materials*, *432*, 128660. https://doi.org/10.1016/j.jhazmat.2022.128660

Liu, S., Zhou, J., Yuan, X., Xiong, J., Zong, M.-H., Wu, X., & Lou, W.-Y. (2024). A dual-mode sensing platform based on metal–organic framework for colorimetric and ratiometric fluorescent detection of organophosphorus pesticide. *Food Chemistry*, *432*, 137272. https://doi.org/10.1016/j.foodchem.2023.137272

Shen, B., Wu, Q., Guo, Y., Qin, J., Chen, H., Yang, Y., Liu, Z., Li, L., Li, W., & Zhu, C. (2025). Modulating the Hydrophilic‐Hydrophobic Microenvironment of MOF‐Stabilized Pt Nanozymes: The Role of H_2_O in the Peroxidase‐Like Catalyzed Reaction. *Advanced Functional Materials*, *35*(8), 2415854. https://doi.org/10.1002/adfm.202415854

Wang, Y., Li, M., Wang, Z., Xu, J., Zhao, J., Gao, Z.-D., & Song, Y.-Y. (2023). Photothermal effect-enhanced peroxidase-like performance for sensitive detection of organophosphorus pesticides on a visual test strip. *Chemical Engineering Journal*, *476*, 146329. https://doi.org/10.1016/j.cej.2023.146329

Yan, X., Zou, R., Lin, Q., Ma, Y., Li, A., Sun, X., Lu, G., & Li, H. (2024). Glutathione‑iron hybrid nanozyme-based colorimetric sensor for specific and stable detection of thiram pesticide on fruit juices. *Food Chemistry*, *452*, 139569. https://doi.org/10.1016/j.foodchem.2024.139569

Zhang, L., Sun, Y., Zhang, Z., Shen, Y., Li, Y., Ma, T., Zhang, Q., Ying, Y., & Fu, Y. (2022). Portable and durable sensor based on porous MOFs hybrid sponge for fluorescent-visual detection of organophosphorus pesticide. *Biosensors and Bioelectronics*, *216*, 114659. https://doi.org/10.1016/j.bios.2022.114659

Zhu, H., Liu, P., Xu, L., Wang, M., Pan, J., & Niu, X. (2022). Emulsion-templated construction of enzyme-nanozyme integrated hierarchically porous hydrogels for smartphone-assisted pesticide biosensing. *Chemical Engineering Journal*, *433*, 133669. https://doi.org/10.1016/j.cej.2021.133669
